# Necroptotic‐susceptible dendritic cells exhibit enhanced antitumor activities in mice

**DOI:** 10.1002/iid3.330

**Published:** 2020-07-14

**Authors:** Zhanran Zhao, Guangzhi Zhang, Yuefang Sun, Astar Winoto

**Affiliations:** ^1^ Department of Molecular and Cell Biology, Cancer Research Laboratory University of California Berkeley California; ^2^ Present address: Guangzhi Zhang, Institute of Animal Sciences of Chinese Academy of Agriculture Sciences Beijing 100193 China

**Keywords:** dendritic cells, FADD, necroptosis, PD‐1

## Abstract

**Introduction:**

Priming of tumor‐specific T cells is a key to antitumor immune response and inflammation, in turn, is crucial for proper T‐cell activation. As antigen‐presenting cells can activate T cells, dendritic cells (DCs) loaded with tumor antigens have been used as immunotherapeutics against certain cancer in humans but their efficacy is modest. Necroptosis is a form of programmed cell death that results in the release of inflammatory contents. We previously generated mice with DC deficiency in a negative regulator of necroptosis, Fas‐associated death domain (FADD), and found that these mice suffer from systemic inflammation due to necroptotic DCs. We hypothesize that FADD‐deficient DCs could serve as a better vaccine than wild‐type (WT) DCs against tumors.

**Materials and Methods:**

FADD‐deficient and WT mouse DCs loaded with the relevant tumor peptide were injected onto mice before or after the syngeneic tumor challenge. DC vaccinations were repeated two more times and anti‐PD‐1 antibodies were coinjected in some experiments. Tumor sizes were measured by caliper, and the percentages of tumor‐free mice or mice survived were examined over time. The cytometric analysis was carried out to analyze various immune populations.

**Results:**

In two separate tumor models, we find that mice receiving FADD‐deficient DCs as vaccine rejected tumors significantly better than those receiving a WT DC vaccine. Tumor growth was severely hampered, and survival extended in these mice. More activated CD8 T cells together with elevated cytokines were observed in mice receiving the FADD‐deficient DC vaccine. Furthermore, we observed these effects were potent enough to protect against tumor challenge postinjection and can work in conjunction with anti‐PD‐1 antibodies to reduce the tumor growth.

**Conclusions:**

Necroptotic‐susceptible DCs are better antitumor vaccines than WT DCs in mice. Our findings suggest that necroptosis‐driven inflammation by DCs may be a novel avenue to generating a strong adaptive antitumor response in the clinical setting.

## Graphical abstract

FADD‐deficient Dendritic cells (DCs) exhibit enhanced antitumor activities in mice. Mice receiving FADD‐deficient DCs post‐tumor challenge exhibited reduced tumor growth and 50% of them survived 19 days after tumor challenge compared to 5% of those receiving wild‐type DCs.

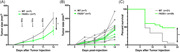

## 1 INTRODUCTION

A fine balance tunes the role of inflammation in the tumor setting. Although appropriate inflammation can be crucial for the proper recruitment and priming of tumor‐reactive T cells, tumors may hijack these same pathways to promote tumor survival, proliferation, and metastasis.^1^, ^2^, ^3^ For example, tumor cells may form an immunosuppressive microenvironment through the recruitment of myeloid‐derived suppressors and co‐opt normal physiological processes driven by inflammation, such as angiogenesis to further tumor progression.^4^ Thus, understanding and manipulating the tumor inflammatory setting are crucial for improving the outcome. Recent studies and clinical trials using combination therapy involving proinflammatory cytokines and vaccines composed of tumor antigens dosed with adjuvants have shown promising results in various tumor models.^5^, ^6^, ^7^ However, much research and novel approaches are still needed.

In the tumor bed, cell death such as necrosis or apoptosis of tumor cells and immune cells can have differential consequences for tumor growth. Manipulating the balance of cell death pathways can, therefore, be a useful approach to stimulating antitumor immunity. Cells undergoing apoptosis are engulfed and removed cleanly by macrophages and thus don't trigger inflammation.^8^, ^9^, ^10^, ^11^ In contrast, cells undergoing a novel cell death pathway, necroptosis, release damage‐associated molecular patterns and are immunogenic.^12^, ^13^, ^14^, ^15^ Necroptosis was first discovered as an alternative necrotic death in cell lines that can be initiated by the Fas death receptor.^16^ In primary cells, those lacking Fas‐associated death domain (FADD), an adapter protein for all the tumor necrosis factor (TNF) family death receptors, or its partner, caspase‐8, are susceptible to necroptosis when triggered by stimuli like TNF, ligands for the Toll‐like receptors or signals through the T‐cell receptor.^12^, ^17^, ^18^, ^19^ Death requires the kinase activity of receptor‐interacting protein 3 (RIP3), a member of the same family as the death domain‐containing kinase RIP1,^20^, ^21^ and the downstream “executioner” protein MLKL.^22^ RIP3 is activated by one of the upstream proteins that contains the RIP homotypic interaction Motif domain like RIP1, Trif, or DAI (ZBP1).^17^, ^23^, ^24^, ^25^ The role of necroptosis in wild‐type (WT) setting is not clear, but the pathway has been suggested as backup immunity for the immune system during infection^12^ or as a developmental checkpoint to eliminate unfit fetuses.^14^ Manipulating the necroptotic pathway may, thus, be a feasible way to enhance anticancer immunity. Indeed, tumor cells undergoing necroptosis have been shown to exhibit potent antitumor activities as an antitumor vaccine.^26^, ^27^

In addition to enhancing T cells to fight cancer using the checkpoint inhibitors and chimeric‐antigen receptors,^2^, ^28^, ^29^, ^30^ the Food and Drug Administration (FDA)‐approved Sipuleucel‐T DC vaccine for human prostate cancer has also been used to increase the tumor immunogenicity.^7^, ^31^, ^32^ However, much improvement is still needed, especially given that the DC vaccine only works modestly.^33^, ^34^ We have previously generated conditional knockout mice lacking the crucial death adaptor protein FADD in the DC compartment through *Cre* expression under the *CD11c* promoter (henceforth, referred to as dcFADD^−/−^ mice).^35^ These mice exhibit a systemic inflammatory phenotype characterized by elevated expression of proinflammatory cytokines including TNF‐α, infiltration of various myeloid populations, and enlarged spleens and lymph nodes.^35^ We demonstrated that these effects were caused by heightened sensitivity of dcFADD^−/−^ dendritic cells to necroptosis. Remarkably, these DCs were not deficient in antigen presentation or T‐cell activation as they exhibited similar ability to stimulate T‐cell proliferation as WT in vitro and in vivo.^35^ We, thus, hypothesized that injection of these dcFADD^−/−^ DCs into tumor‐bearing mice may eventually lead to activation and priming of tumor‐specific T cells to enhance antitumor immunity.

To test our hypothesis, we examined two syngeneic tumor models in mice with various approaches to a therapeutic treatment. We found that dcFADD^−/−^ DCs significantly aided in protection against the tumor through dramatic expansion and activation of host tumor‐specific T cells. We show that this therapy is particularly effective in combination with checkpoint blockade treatment in one tumor model, resulting in complete tumor eradication in some cases and memory response. Thus, we identify a novel approach that has synergy with existing treatments to combat tumor progression.

## 2 MATERIALS AND METHODS

### 2.1 Cell lines

B16 F10‐OVA^36^ and MCA303 cells^37^ were obtained from as kind gifts from Duane Mitchell (Duke University) and Bernard Fox (Providence Portland Medical Center, Portland, OR), respectively. Cells were cultured in complete Dulbecco's modified Eagle's medium supplemented with sodium pyruvate and l‐glutamine (Corning Inc, Corning, NY) and antibiotics. Cells were maintained between 60% and 80% confluence and thoroughly washed with sterile phosphate‐buffered saline (PBS) three times before injection in the indicated amounts. Both were tested mycoplasma negative.

### 2.2 Mice

CD11c‐Cre FADD mice were generated as previously described in the C57BL/6 background.^35^ CD45.1/Thy1.1 WT mice were purchased from Jackson Laboratories. All mice were housed in a specific pathogen‐free facility in Micro‐Isolator cages with autoclaved food. CD11‐Cre positive (dcFADD^−/−^) and negative (WT) in FADD^fl/fl^ allele littermates were used to collect bone marrow‐derived dendritic cells (BMDCs) for the vaccination experiments.

### 2.3 Ethics statement

All the experiments and procedures were performed with the approval of the UC Berkeley Animal Care and Use Committee.

### 2.4 Data availability statement

The data on FADD‐deficient mice have been published before.^35^ FADD floxed mice can be obtained from the Jackson Lab (stock #034740).

### 2.5 DC preparation

BMDCs are prepared using the traditional method with some modifications.^38^ In brief, bone marrow was harvested from 6‐ to 12‐week‐old mice through syringe filtration from femurs. Progenitors cells were cultured in complete Roswell Park Memorial Institute medium supplemented with granulocyte‐macrophage colony‐stimulating factor (GM‐CSF) (1000 U/mL) for 7 days postharvest to allow the generation of dendritic cells. Media was supplemented every two to 3 days. Dendritic cell purity and surface molecular were confirmed by flow cytometry.

### 2.6 Antigen loading

Ovalbumin (OVA) or p15E (KSPWFTTL) peptide (synthesized by Peptide 2.0, Chantilly, VA) was added to BMDC culture 1 day before injection in 50 µg/1 × 10^6^ cells. The peptide was syringed filtered with a 0.2‐µm filter after resuspension in PBS. Labeling was allowed to proceed overnight at 37°C.

### 2.7 Vaccination

Mature DCs were harvested from BMDC culture at 7 days postculture initiation and thoroughly washed and pulsed with appropriate peptide. They were then injected intradermally at the indicated amounts into the abdomen of recipient CD45.1 mice. These injections were repeated every week or 2 to 3 days depending on the therapy schedule. DC health and migration postinjection was tracked by flow cytometry 2 to 3 days postinjection, and animal health was also monitored. Anti‐PD‐1 antibody (BioXCell, West Lebanon, NH) was also injected in some cases as noted in the text.

### 2.8 Tumor challenge

Tumor cells were injected either before DC injection or after vaccination at 1.5 × 10^5^ cells per mouse for B16 F10‐OVA and twice as much for MCA303 tumor cells. Cells were washed in PBS three times before injection subcutaneously on the flank contralateral to the DC injection site. Tumor growth was then monitored with a digital caliper and recorded every 2 days.

### 2.9 Flow analysis

Tumors were harvested 3 to 5 days posttumor injection or when the WT mice grew tumors to 100 mm^3^ in size. Spleen, draining, and nondraining lymph nodes, and the tumor were harvested and red blood cells (RBC) from each were lysed. Tumors were additionally treated with collagenase digestion (1 mg/mL) (Thermo Fisher Scientific, Waltham, MA) and DNase (1 U/mL) (Qiagen, Hilden, Germany) for 25 minutes at 37°C before RBC lysis. Cells were then counted and stained with various surface and intracellular markers. The following antibodies were purchased: Live/Dead stain (Tonbo Biosciences, San Diego, CA), CD4/CD8/CD44/CD62L (BioLegend, San Diego), interferon‐γ (IFN‐γ)/TNF‐α/FoxP3 (BD Biosciences, San Jose, CA). For tetramer staining, incubation was performed at room temperature and cells were immediately analyzed by flow cytometry. For all other staining, cells were stained on ice and fixed with either BD Cytofix or eBiosciences Transcription Factor Staining Kits according to manufacturer protocols and then analyzed within the next 2 days.

### 2.10 Statistics

Statistical analysis was performed using the GraphPad Prism software. *P *< .05 was considered significant. Comparisons were made using a two‐way analysis of variance for tumor size and logrank Mantel‐Cox test for tumor‐free mice and percent mouse survival.

## 3 RESULTS

### 3.1 FADD‐deficient DCs provide better protection than WT DCs against B16‐F10‐OVA challenge in a vaccine model

**Figure 1:**
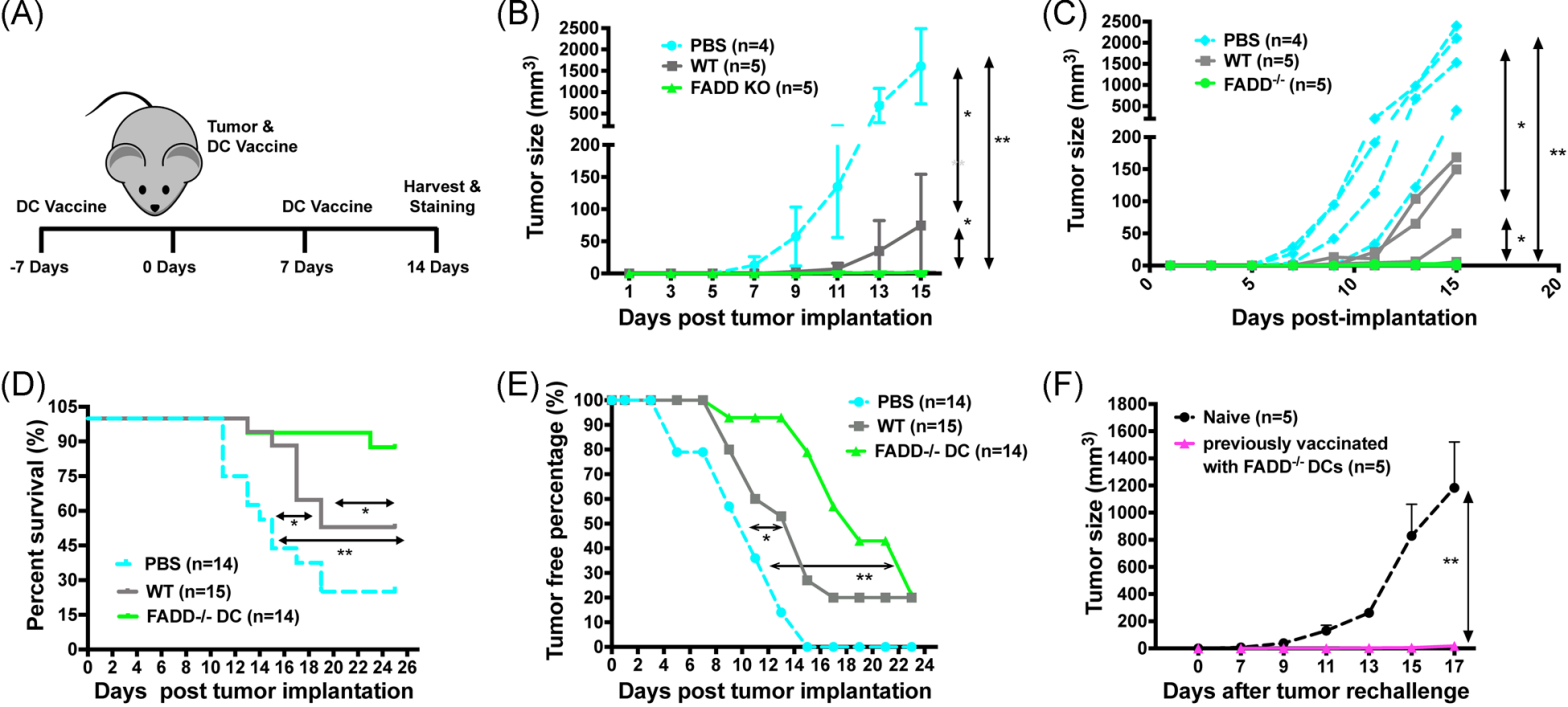
Vaccination with peptide‐pulsed FADD‐deficient DCs protects against B16 tumor challenge. A, Vaccination schedule. Bone marrow‐derived DCs from CD45.2 wild‐type (WT) or dcFADD^−/−^ mice were stimulated with granulocyte‐macrophage colony‐stimulating factor in culture for 7 days. They were then pulsed with ovalbumin (OVA) protein overnight before intradermal injection into CD45.1 B6 mice in the abdomen at the indicated time points. B, Average tumor sizes of CD45.1 mice after subcutaneous injection of B16 F10 OVA tumors and subsequent vaccination with PBS (n = 4), WT DCs (n = 5), or FADD^−/−^ DCs (n = 5). Volumes were calculated according to the modified ellipsoid formula of 1/2 x L x W x W, where L = length and W = width of the tumor. Data are representative of three independent experiments. C, Individual tumor growth curves corresponding to the graph in (B). D, Survival of tumor‐injected mice postvaccination with PBS (n = 14), WT DCs (n = 15), or FADD^−/−^ DCs (n = 14). Data are a compilation of three independent trials. Endpoint was defined as tumor size exceeding 1.5 cm in any direction or ulceration. E, Proportion of mice with no palpable tumors postvaccination with PBS (n = 14), WT DCs (n = 15), or FADD^−/−^ DCs (n = 14). Data are a compilation of three independent trials. Palpable tumors were defined as visible as distinct masses on the skin and able to be precisely measured by a digital caliper. F, Mice previously vaccinated with FADD^−/−^ DCs (n = 5) 2 weeks earlier were subjected to a new round of tumor injection. Mice (n = 5) that have not previously vaccinated were used as control (naive). Data are representative of two independent experiments. *P < .05, **P < .01 (two‐way analysis of variance for (B and E) and logrank Mantel‐Cox test for (C and D)). DC, dendritic cell; FADD, Fas‐associated death domain; KO, knockout; PBS, phosphate‐buffered saline

To see if FADD‐deficient DCs can protect mice from tumor challenge better than WT DCs, we first used the B16‐F10‐OVA subcutaneous model. Following published protocol,^36^ we injected OVA‐pulsed WT (FADD^fl/fl^) or dcFADD^−/−^ BMDCs from CD45.2 C57BL/6 mice intradermally into CD45.1 C57BL/6 recipient mice three times at 1‐week intervals (Figure 1A). One week after the initial DC vaccination, we subcutaneously injected B16‐F10‐OVA tumor cells at the contralateral site to the initial DC injection. Tumor sizes were measured by caliper and the percentages of tumor‐free mice or mice survived were examined over time. Although the WT DCs still offered some measure of protection as noted by others,^36^ the dcFADD^−/−^ DCs significantly delayed the onset of tumor growth and in some cases offered complete protection against the tumor challenge by the endpoint (tumor size exceeding 1.5 cm at any direction or showing ulceration) of the WT group (Figure 1B‐D). By day 11, most of the mice in the PBS control group had grown tumors of around 100 mm^3^, whereas those that received WT DCs have just begun to present with black spots indicative of early tumor growth. As B16 melanoma cells proliferate rapidly in a subcutaneous model, some tumors in the PBS control and WT groups had already reached or were close to the endpoint when we first noted small tumors in the dcFADD^−/−^ group. Indeed, more than 90% of the dcFADD^−/−^ group remained tumor‐free at 2 weeks postinjection, while the PBS and WT groups had only 20% and 50% tumor‐free mice, respectively (Figure 1E). Upon rechallenge, mice that have previously been vaccinated with dcFADD^−/−^ DCs showed complete tumor rejection, suggesting a memory is provided by this treatment (Figure 1F).

### 3.2 Tumor protection is accompanied by the expansion of tumor‐specific CD8 T cells

**Figure 2:**
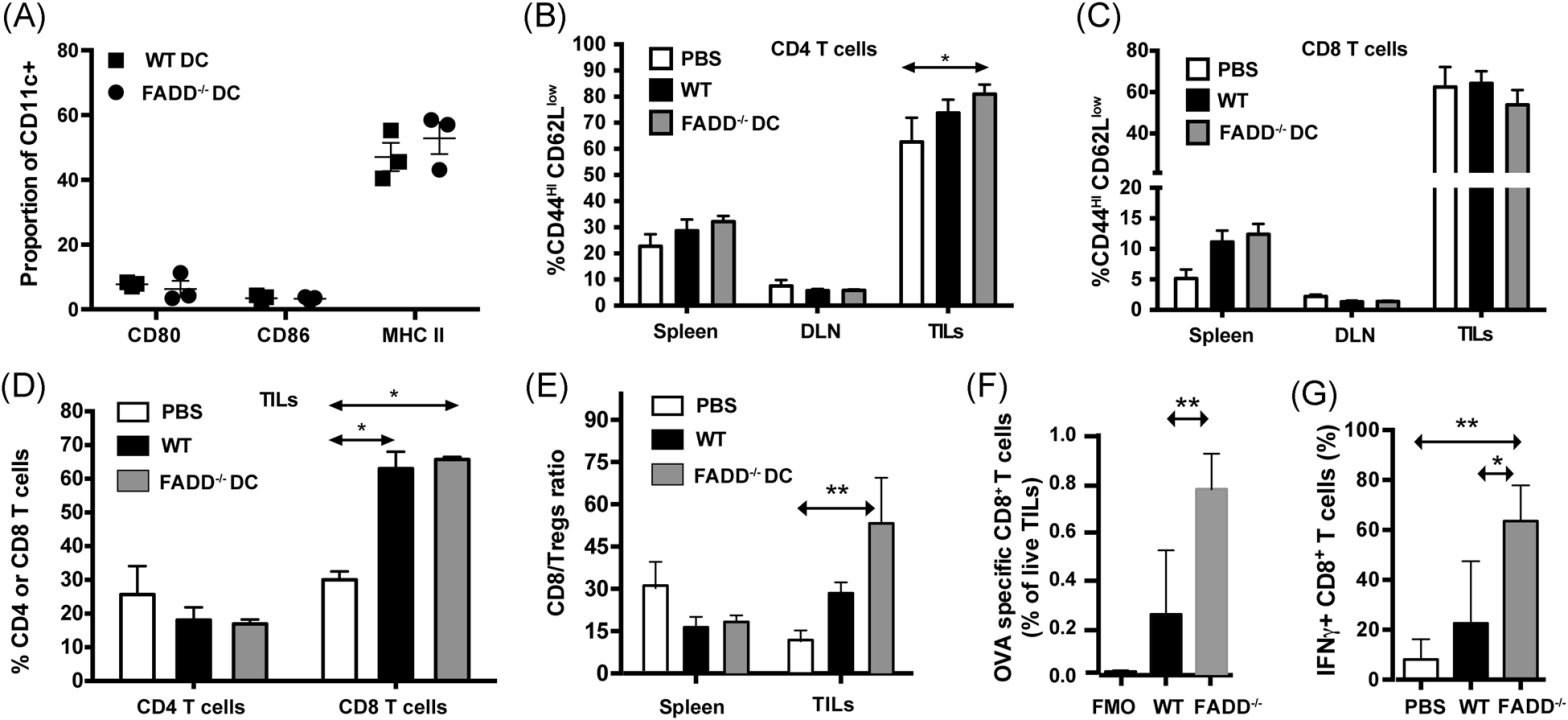
Tumor protection from the dcFADD^−/−^ DC vaccine is primarily driven by CD8 T cells. Mice vaccinated per schedule outlined in Figure 1A were analyzed by flow cytometry 2 weeks after tumor injection. A, Flow cytometry of activation markers on WT‐ and FADD‐deficient DCs on draining lymph nodes 2 weeks after tumor injection. Gating was as follows: single cells > live events > CD45+, B220− events > CD3− events > CD11b+, CD11c+ events. B, CD4 and (C) CD8 T‐cell activation as measured by CD44^high^/CD62L^low^ staining in the spleen, draining lymph nodes (DLN), and tumor‐infiltrating lymphocytes (TILs). Gating was as follows: single cells > live events > CD45+, B220− events > CD3+ events > CD4 or CD8+ events. D, Proportion of CD4+ and CD8+ T cells in TILs two weeks postinjection. Gating was as follows: single cells > live events > CD45+, B220− events > CD3+ CD4+ or CD3+ CD8+ events. E, Tregs were identified by FoxP3 expression, as revealed by intracellular staining with eBiosciences Transcription Factor Kit. The ratio was expressed as the number of CD8 T cells per 1 Treg. F, Tetramer staining with ovalbumin (OVA)/K^b^ tetramer was performed at room temperature, with preincubation with tetramer before the addition of other surface stains. The number of OVA‐specific T cells was quantified as a proportion of the total population of live TILs; fluorescent minus one controls. Gating was as follows: single cells > live events > CD45+, B220− events > CD3+ events > CD8+ events > OVA/K^b^ tetramer. G, Interferon‐γ (IFN‐γ) production was measured by intracellular staining after blocking of TIL samples with GolgiPlug/GolgiStop for 4 hours. Cells were permeabilized and fixed with BD Cytofix/Cytoperm. Gating was as follows: single cells > live events > CD45+, B220− events > CD3+ events>CD8+ events > IFN‐γ. B‐E and G, Data were collected from four mice for the PBS control and four mice for the WT DCs in four individual experiments and from three mice for FADD^−/−^ DCs in three individual experiments. A and F, Data were collected from three mice for WT DCs and three mice for FADD^−/−^ DCs in three individual experiments. *P < .05, **P < .01 (two‐way analysis of variance). DC, dendritic cell; FADD, Fas‐associated death domain; MHC, major histocompatibility; PBS, phosphate‐buffered saline

**Figure 3:**
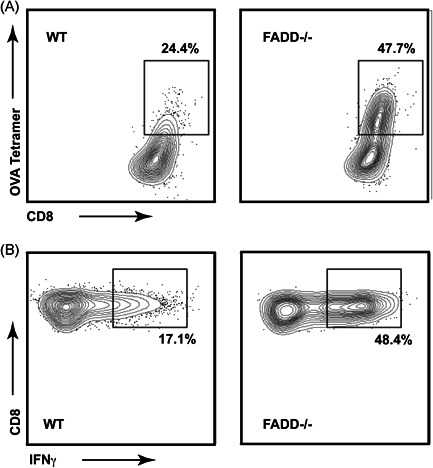
Representative flow cytometric plots for ovalbumin (OVA) tetramer and IFN‐γ staining. Mice vaccinated per schedule outlined in Figure 1A and challenged by B16‐OVA were analyzed by flow cytometry 2 weeks after tumor injection. A, Representative plots of the OVA/K^b^ tetramer staining of the TILs receiving wild‐type (WT) or FADD^−/−^ dendritic cells (DCs) corresponding to data in Figure 2F. B, Representative plots of the IFN‐γ staining of the TILs receiving WT or FADD^−/−^ DCs corresponding to data in Figure 2G. Gating for both (A and B) was as follows: single cells > live events > CD45+, B220− events > CD3+ events > CD8+ events. FADD, Fas‐associated death domain; IFN‐γ, interferon‐γ; TIL, tumor‐infiltrating lymphocyte

We examined the splenic, lymph node, and tumor‐infiltrating lymphocyte (TIL) populations of these mice posttumor injections to determine the factors that lead to this protection. We found no differences in DC activation or changes in the numbers or proportions of myeloid populations in the draining and nondraining lymph nodes. CD80/CD86 and MHC Class II expression levels were similar across all mice examined (Figure 2A). We also failed to observe any differences between the WT and dcFADD^−/−^ DC injected groups in terms of T‐cell activation or overall T‐cell numbers in the TILs (Figure 2B,C). However, we did observe a specific increase in the proportion of CD8 T cells in the TILs of both WT and FADD‐deficient DC‐treated groups over PBS controls (Figure 2D) as well as an increase in the CD8 to Treg ratio (Figure 2E), an indicator that has previously been associated with a strong antitumor response in reaction to immunotherapy.^39^ Among the CD8+ T cells in TILs, mice receiving FADD^−/−^ DC vaccine had a further increase of OVA‐specific T cells and elevated IFN‐γ expression over those receiving WT vaccine (Figures 2F,G and 3). These data point to the generation of a potent T‐cell‐driven antitumor response in mice receiving the FADD‐deficient DC vaccine.

### 3.3 Treatment of tumors postimplantation with dcFADD^−/−^ DCs in combination with PD‐1 antibody can slow and defend against tumor progression

**Figure 4:**
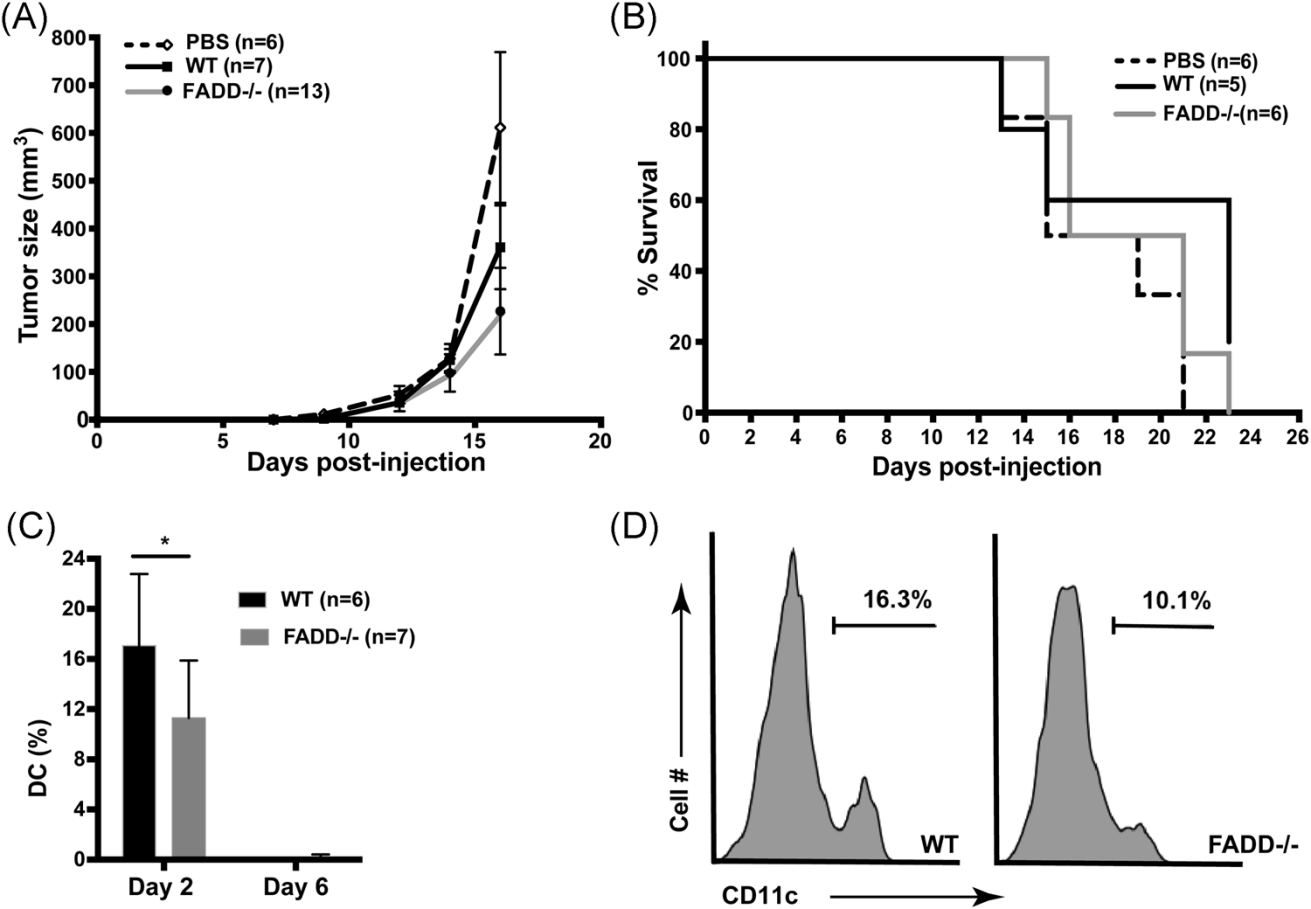
FADD‐deficient DCs in posttumor studies and their presence in the lymph nodes after injection. A, Tumor sizes of PBS (n = 6), WT DCs (n = 7), or FADD‐deficient DC (n = 13) treated only mice. Tumors were allowed to grow for at least 3 days before injection every 3 days for three times with peptide‐pulsed DCs. Data are representative of three independent experiments. B, Survival of mice treated with B16‐OVA tumors and injected with PBS (n = 6), WT DCs (n = 5), or FADD‐deficient DC (n = 6) 3 days later and two more times at days 6 and 9. Data are representative of three independent experiments. C, *FADD^−/−^* or wild‐type (WT) CD45.2^+^ DCs obtained from bone marrows of littermates were injected subdermally into CD45.1 B6 mice. Two or six days later, the percentage of CD45.2 CD11c DCs from the draining lymph nodes was measured by flow analysis (n = 5 for day 2 and n = 7 for day 6 for both WT and FADD^−/−^ DCs). The experiment has been repeated one more time with similar results. D, Representative flow cytometric plots for the experiments in (C). Gating for (C and D) was as follows: single cells > live events > B220− events > CD3− events > CD45.2+ events > CD11b+, CD11c+ events. *P < .05 (two‐way analysis of variance (A and C); logrank Mantel‐Cox test for (B)). DC, dendritic cell; FADD, Fas‐associated death domain; PBS, phosphate‐buffered saline

As preinjection tumor vaccines do little to reflect the actual clinical setting, we sought to evaluate the potency of this treatment as a therapeutic vaccine after tumor injection. We, thus, first injected B16‐F10‐OVA cells subcutaneously and waited at least 3 days before subsequent injection with three rounds of dcFADD^−/−^ dendritic cells. Using this treatment schedule, we saw no significant protection afforded by the dcFADD^−/−^ DCs compared to control (Figure 4A). Tumors grew at the same rate in all groups as measured by caliper, markedly different from the delay in tumor onset we previously observed in the vaccine model. Almost all mice reached experimental endpoints at similar time points, about 2 weeks after initial tumor implantation (Figure 4B).

As we observed a lack of protection by DCs posttumor injection, we wondered whether a shorter time span of injection is warranted. To gauge the lifespan and migration patterns of dcFADD^−/−^ DCs postinjection, we examined both host and recipient DC populations at various time points after DC intradermal injection (without tumor challenge). Although we didn't induce necroptosis in the injected *FADD* ^−*/*−^ DCs, we found slightly fewer *FADD* ^−/−^ donor DCs (CD45.2^+^) in the draining lymph nodes when compared to WT DCs at day 2 postinjection (Figure 4C,D). The percentages of donor DCs detected continued to drop at later days and at day 6 postinjection, both WT and FADD‐deficient DCs could not be found (Figure 4C).

**Figure 5:**
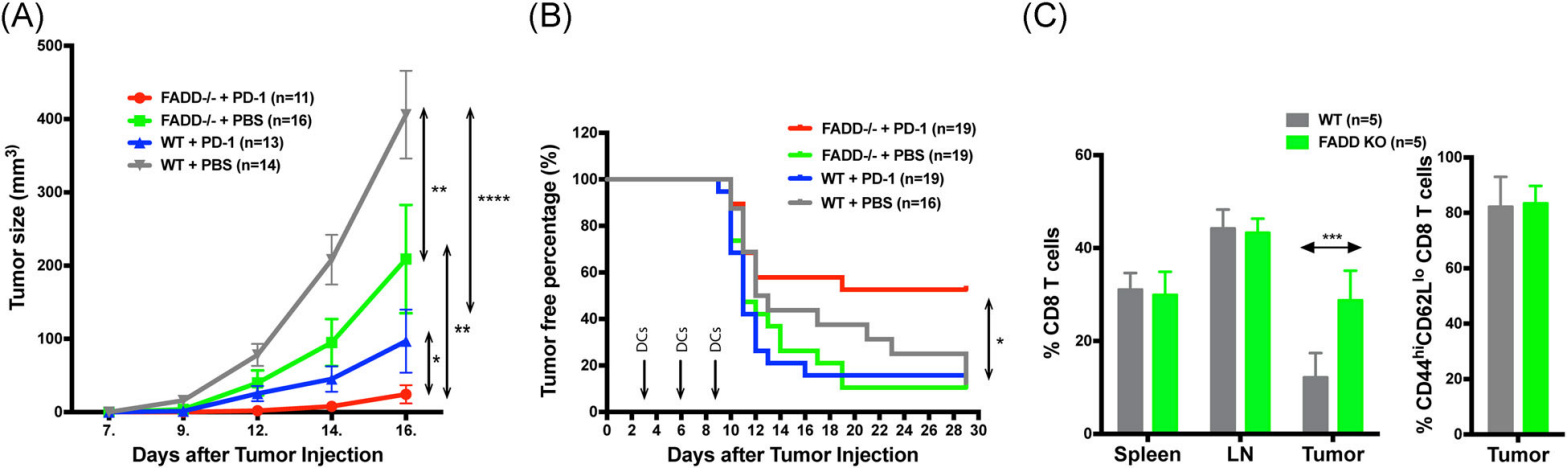
Combination therapy with anti‐PD‐1 results in a protection against established tumors. A, Tumor sizes of mice treated with combination therapy (WT‐ or FADD‐deficient DC with or without anti‐PD‐1 antibodies). As before, tumors were allowed to grow for 3 days before injection with DCs. A 100 μg of PD‐1 antibody (BioXCell clone RPM1‐14) or PBS control were simultaneously injected intraperitoneally. This treatment was repeated three times at 3‐day intervals. Data are a combination of three independent experiments (n = 11 for FADD^−/−^ DCs and anti‐PD‐1, n = 16 for FADD^−/−^ DCs and PBS, n = 13 for WT DCs and anti‐PD‐1, and n = 20 for WT DCs and PBS). B, Tumor‐free proportions of mice treated with combination therapy of WT‐ or FADD‐deficient DC with or without anti‐PD‐1 antibodies. Tumor free defined as tumors smaller than 50 mm in diameter or otherwise not measurable by a caliper. DC injections were denoted as arrows. Data are a combination of three independent experiments (n = 19 for FADD^−/−^ DCs and anti‐PD‐1, n = 19 for FADD^−/−^ DCs and PBS, n = 19 for WT DCs and anti‐PD‐1, and n = 16 for WT DCs and PBS). C, Left panel: the proportion of CD8 T cells in the spleen, draining lymph nodes (LN), and tumor‐infiltrating lymphocytes (tumor). Right panel: activated CD8 T cells in tumor‐infiltrating lymphocytes (tumor) as measured by CD44^high^/CD62L^low^ staining. Gating was as follows: single cells > live events > CD45+, B220− events > CD3+ events > CD8+ events for the left panel and additional gating > CD44^hi^/CD62L^lo^ for the right panel. Data were collected from five mice for WT DCs and five mice for FADD^−/−^ DCs from two individual experiments. *P < .05, **P < .01,***P < .001, ****P < .0001 (two‐way analysis of variance (A); logrank Mantel‐Cox test (B)). DC, dendritic cell; FADD, Fas‐associated death domain; KO, knockout; PBS, phosphate‐buffered saline; WT, wild‐type

As donor DCs disappear after 6 days, we reasoned that the treatment schedule of three times DC injections spaced only 3 days apart was appropriate. Indeed, sometime we were able to detect significant differences between dcFADD^−/−^ and WT groups in the growth of tumors but still not the survival or the proportions of mice that remained tumor‐free (Figure 5A,B). As T cells are the major contributing factor to the tumor protection phenotype we observe,^40^, ^41^ and PD‐1 antibody treatment has been shown to be effective against mouse and human tumors in combination with other therapies,^42^, ^43^, ^44^ we sought to combine DC injection with anti‐PD‐1 treatment. In these experiments, we allowed tumor cells to grow out as before and performed three rounds of DC injections every 3 days intradermally, contralateral to the tumor implantation site, along with three doses of intraperitoneal PD‐1 antibody injection at the same time. With this combination treatment, we observe a dramatic increase in the survival and proportion of tumor‐free mice (Figure 5A,B). Approximately, 50% of the dcFADD^−/−^ + PD‐1 antibody injected group did not show any observable tumors over three separate replicates, compared to 10% to 20% for those that received only DCs or WT DCs + PD‐1 treatments (Figure 5B). Tumor volumes were also reduced in those mice that received the combination therapy, with some only one fourth to one half as large 2 weeks postimplantation of the tumor (Figure 5A). Examining the TILs, we observed increased numbers of elevated CD8 proportions in those receiving FADD^−/−^ DCs (Figure 5C), though we do not note changes in the proportions of activated cells as shown by CD44/CD62L staining (Figure 5C). Upon rechallenge, mice that have previously been vaccinated with dcFADD^−/−^ DCs and anti‐PD‐1 antibody showed no tumor growth after more than 3 weeks (6/6), suggesting a long‐term memory response provided by this treatment. We concluded that a combined treatment of anti‐PD‐1 antibodies and FADD‐deficient DCs can significantly protect mice from tumor challenge in a synergistic pattern.

### 3.4 FADD‐deficient DCs provides better tumor protection than WT DCs in a separate tumor model

**Figure 6:**
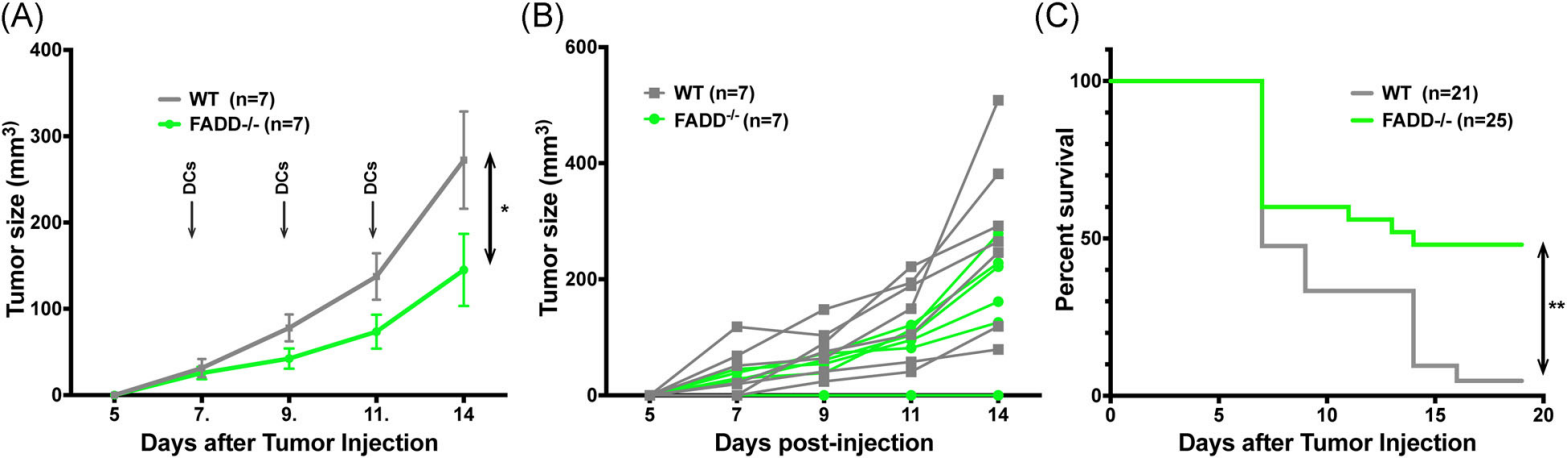
FADD‐deficient DCs alone can protect against established MCA303 tumors. A, MCA303 tumor cells were injected subcutaneously at day 0. Three or seven days later, p15E peptide loaded WT (n = 7) or FADD^−/−^ (n = 7) DCs were injected. This was followed by two more rounds of the WT or FADD^−/−^ DC injections. Tumor sizes were measured by caliper. DC injections were denoted as arrows. Data are representative of three independent experiments. *P < .05 (two‐way analysis of variance). B, Individual tumor growth curves corresponding to the graph in (A). C, Survival of MCA303 tumor‐injected mice. Mice were injected with MCA303 tumor cells at day 0, followed by two to three times injections of p15E‐loaded WT DCs (n = 21) or FADD^−/−^ DCs (n = 25) 3 or 7 days later. The endpoint was defined as tumor size exceeding 1.5 cm in any direction or ulceration. Data are compilation of three independent experiments. *P< .05, **P < .01 (two‐way analysis of variance (A); logrank Mantel‐Cox test (C)). DC, dendritic cell; FADD, Fas‐associated death domain; WT, wild‐type

To see if *FADD* ^−/−^ DCs vaccine works in another syngeneic tumor model and by using a more physiological antigen for vaccines, we employed MCA303 sarcoma. Many H2^b^ haplotype tumor models like B16, MC38 and methylcholanthrene‐induced fibrosarcoma (like MCA303) preferentially express the *env* transcript of murine leukemia virus.^45^, ^46^, ^47^ These tumors can induce CD8^+^ T‐cell response to the p15E *env* epitope in an H‐2 K^b^ restricted fashion.^48^, ^49^ The p15E peptide‐loaded WT DCs have been shown to partially protect mice from B16 tumors.^45^ We, thus, used the p15E env peptide in the DC vaccine. We injected a large dose of MCA303 cells followed by two to three rounds of subdermal injections of p15E‐loaded dcFADD^−/−^ DCs starting at day 3 or day 7 after initial tumor injection. We saw protection against tumor challenge, with significant differences in tumor size between mice receiving FADD‐deficient DCs and WT DCs (Figure 6A,B). Moreover, almost 50% of the mice receiving dcFADD^−/−^ DCs survived 19 days after tumor challenge compared to 5% of those receiving WT DCs (Figure 6C). As has been shown by others,^50^, ^51^ anti‐PD‐1 antibodies have significant effects against weakly immunogenic tumors such as MCA303; anti‐PD‐1 alone suppressed MCA303 growth completely, and thus, no additional effects could be seen with a combination of WT or FADD‐deficient DCs.

## 4 DISCUSSION

Our results demonstrate that FADD‐deficient DCs can promote a strong T‐cell‐dependent antitumor response that is effective against two tumor models. FADD is a negative regulator of RIPK3, its absence can lead to the activation of RIPK3‐MLKL‐dependent necroptosis,^12^, ^13^, ^14^, ^15^ and the subsequent release of inflammatory contents. We did not induce necroptosis before DC injection and DCs are not undergoing cell death in culture, we hypothesize that the tonic level of TNF‐α in the recipient mice may trigger necroptosis in the DCs in vivo or that migration of DCs to the draining lymph nodes is affected. We consider the latter possibility to be unlikely because we have previously shown that injection of SIINFEKL pulsed *FADD* ^−/−^ DCs into footpads of *beta2m* ^−*/*−^ mice can stimulate adoptively transferred OT‐1 T cells to the same degree of that of WT DCs.^35^ We have shown that FADD‐deficient DCs are sensitive to TNF‐mediated necroptosis in vitro.^35^ This hypothesis is supported by our observation that we saw significantly lower numbers of dcFADD^−/−^ DCs in vivo 2 days after injection compared to WT DCs in the draining lymph nodes. However, future experiments using dcFADD^−/−^/*TNF‐RI* ^−/−^ combined knockouts would be necessary to prove our hypothesis. Alternatively, other unknown cytokines may induce FADD‐deficient DCs to undergo necroptosis in vivo. In addition to necroptosis mediated by RIPK3, the absence of FADD may also activate the cell‐death independent function of RIPK3. Several groups have shown that RIPK3 can promote inflammation independent of its role in cell death.^52^, ^53^, ^54^ Thus, the activation of RIPK3 in FADD‐deficient DCs may increase inflammation indirectly through necroptosis or directly through RIPK3‐dependent cytokine production.

The incredibly potent antitumor effects provided by prevaccination with dcFADD^−/−^ alone, which is reminiscent of original B16 GM‐CSF + anti‐CTLA‐4 trials,^55^ shows that this treatment allows the effective generation of an effective T‐cell response before the tumors are even allowed to replicate. The increase in tumor‐specific CD8 numbers and activity also seems to corroborate this view. Several groups have previously shown that cancer DC vaccines are mediated by endogenous DCs.^56^, ^57^ Among the two DC populations, cDC1 (XCR1^+^, CD8^+^, and CD103^+^) and cDC2 (CD172^+^),^58^ cDC1 has been shown to be important for cross‐presentation and is essential for immunogenicity of necroptotic cells^26^ as well as antitumor immunity.^59^ However, DCs from dcFADD^−/−^ mice contain fewer CD103^+^ cells and CD8^+^ DCs (ie, cDC1).^35^ It is, thus, possible that necroptosis of FADD‐deficient DCs may stimulate the activation of the endogenous DCs that then increase antitumor immunity. As we detect no significant differences in the activation state of the injected or host DCs by conventional markers, how this occurs is still unresolved. Nevertheless, our data suggest that FADD‐deficient DCs can help to generate an antitumor inflammatory microenvironment that enhances the activation of T cells to clear the tumor.

As necroptosis is a relatively rapid event and the release of inflammatory molecules is short‐lived, our original experiments involving week‐long posttumor injections of DCs may have missed the critical timing for the generation of the T‐cell response. This observation suggests the requirement for a healthy DC population to already be present near the tumor site or in the draining lymph node, which may not always be the case in a clinical setting. Thus, combination therapy as we have done here with checkpoint blockade inhibitors is likely necessary to prolong the effect of the treatment. Indeed, in mice receiving this combination therapy, roughly half see their tumors disappear altogether and also retain a memory response several weeks after treatment.

The fact that we are able to extend these findings to two models suggests that these necroptotic DCs may be able to generate potent inflammatory responses that are universal and can override tumor suppression mechanisms. This effect is most likely systemic as we injected DCs contralateral to the site of tumor injections. In both models, high amounts of tumor‐specific antigen are required to generate the response, as incubation with whole‐cell extract from the tumor cell lines alone^60^ was not strong enough to provide protection (unpublished data). Thus, identification of tumor neoantigens may be critical for this therapy to be effective in the clinic to treat human cancer. The strategy of DC vaccines loaded with the patients’ tumor neoantigens might be a promising new approach to fight cancer. Indeed, increased T‐cell antitumor activities were seen in 3 melanoma patients receiving DC vaccines with their tumor neoantigens.^7^ On the contrary, the FDA‐approved Sipuleucel‐T DC vaccine uses ex vivo blood DCs loaded with a prostate antigen. Based on our data, we propose that its efficacy might be enhanced if DCs are rendered susceptible to necroptosis by deleting the endogenous *FADD* gene through CRISPR methodology.^61^, ^62^ Alternatively, c‐FLIP_S_, which has previously been shown to confer susceptibility to necroptosis,^63^, ^64^ can be introduced into patients’ DCs through a lentiviral vector. In any case, we demonstrate a novel approach that leads to effective T‐cell responses against tumors in mice with potential future applications in humans.

## CONFLICT OF INTERESTS

The authors declare that there are no conflict of interests.
